# Publication of Datasets, a Step toward Advancing Data Science

**DOI:** 10.1016/j.xops.2023.100381

**Published:** 2023-08-11

**Authors:** Emily Y. Chew

**Affiliations:** Bethesda, Maryland

Data science is one of several foci of *Ophthalmology Science*, the latest journal to join the American Academy of Ophthalmology journal family. The inaugural special issue of *Ophthalmology Science* was devoted to artificial intelligence and big data/data science.^1^ Data science is the study of data to extract insights, with a multidisciplinary approach, from the fields of mathematics, statistics, artificial intelligence, and computer science. The process starts with the collection of data, whether from structured or unstructured sources. The data are analyzed using several approaches that include descriptive, predictive causal, and prescriptive analytics and machine learning for diagnostic and outcome prediction. The success of data science relies on the availability of a large volume of data that are varied, and representative of the population under investigation.

Why is it imperative to have access to datasets, especially those that are well curated? Data are the backbone of all research, especially those involving data science/machine learning. These curated datasets provide great value because other investigators may use these data to formulate their own hypotheses that may be different from those proposed by the researchers who had assembled the dataset. Other investigators may use them for a variety of purposes, including validating their own research by developing algorithms. Data, both text and images, may come from electronic medical records, epidemiologic studies, and randomized clinical trials. Using a variety of data that are highly curated, hypothesis generation, evaluation of outcomes, and other research activities will be accelerated. Researchers often do not have access to high-quality datasets. It is the hope that the research community will share in both highly curated and consistently structured datasets to provide important datasets to move this field forward. Harmonization of both data collection and structure of the datasets would also enhance the quality of these datasets.

Data are the backbone of all research, especially those involving data science/machine learning.

Bilton et al^2^ publish, in this issue of *Ophthalmology Science*, a dataset that focuses on retinal vein occlusions and associated systemic diseases of major cardiovascular events from INSIGHT.^2^ INSIGHT is led by the Moorfields Eye Hospital National Health Service Foundation Trust, working in partnership with the University Hospitals of Birmingham National Health Service Foundation Trust UK to collect data from electronic patient records with a focus on eye health. However, the analyses of these data could lead to discoveries in other diseases such as cardiovascular, diabetes, and dementia. Their goal is to provide large, anonymized sets of patients for data science research.

In this particular dataset on retinal vein occlusions, the data were collected from the hospital’s electronic patient records, which primarily included structured data from the eye examination, including OCT images and data from the medical work-up performed to determine the presence of cardiovascular disease. This report summarized the content of the dataset, how it was curated, and its potential uses. The report datasheet had a list of all the variables that were available for the phenotyping of the retinal disease, including a complete history of medication use, major adverse cardiovascular events, and ocular and cardiac procedures. Additional data included a number of other procedures that were conducted to investigate many other organ systems. The data are available from INSIGHT to researchers through a structured application process.

Similarly, another dataset from INSIGHT also appears in this issue of *Ophthalmology Science*. Kale et al^3^ describe the data collected from diabetic retinopathy screening from the Birmingham, Solihull, and Black Country Eye Screening Program. They consist of 6 202 161 anonymized images and linked screening data from 246 180 patients starting from January 1, 2007. All persons with diabetes ≥ 12 years of age attended annual digital retinal photography and the patients’ diabetic status and visual acuity data are available. The dataset descriptor also summarized the content, curation method, and potential uses of the data. Data are available through a structured application process (https://www.insight.hdrhub.org/).

So why should datasets be published in a peer-reviewed journal? Publication of available datasets is an important service to the research community and for advancement in data science. Data are valuable research resources that are not readily available or even visible to researchers. Publication in a peer-reviewed journal provides a framework for presenting data that have been vetted by a thorough review process with rigorous standards. Publication in an open access journal would readily increase the availability and use of the dataset. An added benefit for the authors includes the fact that they will have fulfilled the requirement imposed by funding agencies for data sharing. Furthermore, the authors have a citable publication in a reputable journal that would serve as a potential incentive for researchers to publish and share their datasets.

In 2020, a review was conducted to identify all publicly available ophthalmic imaging datasets, and to create a central directory of the available datasets for access, because there was no centralized directory of ophthalmic datasets. There was also little knowledge regarding the size and number of imaging datasets that were publicly available.^4^ The authors discussed the barriers to access, usability, and generalizability of the data. The results showed that 94 datasets were accessible, and the report provided the list of accessible datasets. This exercise was repeated in 2023 by another group of researchers who focused on color fundus images, and they noted still “only a few places to store or list ophthalmic datasets, and many of them are hard to find, making it hard to obtain high-quality, representative data useful for a given purpose.”^5^ These researchers generated a central list of 120 accessible datasets with color fundus images.

Clearly, there is not only a need to improve our collection of datasets, but to also increase their visibility and accessibility for all researchers. *Ophthalmology Science* welcomes the submission of such detailed descriptions of datasets from a variety of sources, including clinical trials, population-based studies, electronic medical records, basic science laboratory findings, genomic data, and all ophthalmic images including functional test images. Although this is an open access journal, for the purpose of improving our ability to perform studies to advance data science, we will waive article processing fees for these dataset reports. The manuscripts will undergo a rigorous review process for the completeness of the data and to confirm that the appropriate descriptors are provided. The methods for application for access to these datasets should also be clearly described. These reports that summarize the data must be cited by all investigators who utilize these datasets. Because ophthalmic images are often a component of the datasets, there is a mixed response from various academic institutions as to whether retinal images are considered biometric or personally identifiable information. This may result in some datasets having controlled access rather than open access. *Ophthalmology Science* would welcome all datasets with both controlled and open access. However, we do acknowledge the leader in this field, *Translational Vision Science & Technology* (TVST), has already established a central directory for those datasets that are open source.^6^ The establishment of dataset publications within *Ophthalmology Science* may indeed complement the work of TVST. Sharing our datasets with our colleagues will no doubt increase our innovation, creativity, and productivity in the field of data science.
